# A hERG mutation E1039X produced a synergistic lesion on *I*_Ks_ together with KCNQ1-R174C mutation in a LQTS family with three compound mutations

**DOI:** 10.1038/s41598-018-21442-6

**Published:** 2018-02-15

**Authors:** Jie Wu, Yuka Mizusawa, Seiko Ohno, Wei-Guang Ding, Takashi Higaki, Qi Wang, Hirohiko Kohjitani, Takeru Makiyama, Hideki Itoh, Futoshi Toyoda, Andrew F. James, Jules C. Hancox, Hiroshi Matsuura, Minoru Horie

**Affiliations:** 1 Department of Pharmacology, Medical School of Xi’an Jiaotong University, Xi’an, Shaanxi 710061 China; 2 Department of Cardiovascular and Respiratory Medicine, Shiga University of Medical Science, Shiga, Japan; 3 Department of Physiology, Shiga University of Medical Science, Shiga, Japan; 4 Department of Pediatrics, Ehime University School of Medicine, Ehime, Japan; 5 Department of Cardiovascular Medicine, Kyoto University Graduate School of Medicine, Kyoto, Japan; 6 School of Physiology, Pharmacology and Neuroscience, University of Bristol, Bristol, United Kingdom

## Abstract

Congenital long QT syndrome (LQTS) caused by compound mutations is usually associated with more severe clinical phenotypes. We identified a LQTS family harboring three compound mutations in different genes (*KCNQ1*-R174C, *hERG*-E1039X and *SCN5A*-E428K). *KCNQ1*-R174C, *hERG*-E1039X and *SCN5A*-E428K mutations and/or relevant wild-type (WT) cDNAs were respectively expressed in mammalian cells. *I*_Ks_-like, *I*_Kr_-like, *I*_Na_-like currents and the functional interaction between KCNQ1-R174C and hERG-E1039X channels were studied using patch-clamp and immunocytochemistry techniques. (1) Expression of KCNQ1-R174C alone showed no *I*_Ks_. Co-expression of KCNQ1-WT + KCNQ1-R174C caused a loss-of-function in *I*_Ks_ and blunted the activation of *I*_Ks_ in response to isoproterenol. (2) Expression of hERG-E1039X alone and co-expression of hERG-WT + hERG-E1039X negatively shifted inactivation curves and decelerated the recovery time from inactivation. (3) Expression of SCN5A-E428K increased peak *I*_Na_, but had no effect on late *I*_Na_. (4) *I*_Ks_ and *I*_Kr_ interact, and *hERG*-E1039X caused a loss-of-function in *I*_Ks_. (5) Immunocytochemical studies indicated that KCNQ1-R174C is trafficking defective and hERG-E1039X is defective in biosynthesis/degradation, but the abnormities were rescued by co-expression with WT. Thus, *KCNQ1*-R174C and *hERG*-E1039X disrupted *I*_Ks_ and *I*_Kr_ functions, respectively. The synergistic lesion, caused by *KCNQ1*-R174C and *hERG*-E1039X in *I*_Ks_, is very likely why patients showed more severe phenotypes in the compound mutation case.

## Introduction

Congenital long QT syndrome (LQTS) is a life-threatening condition characterized by an abnormally prolonged QT interval on the electrocardiogram (ECG) and *torsades de pointes (TdP)*-triggered cardiac events such as syncope, cardiac arrest and sudden cardiac death^1,2^. Molecular genetic studies have revealed that congenital LQTS is linked to mutations in genes encoding for cardiac ion channels or their regulatory proteins. To date at least 15 genes have been identified to be responsible for different subtypes of the syndrome^3,4^, with the first three LQTS (LQT1-3, caused by mutations in *KCNQ1*, *KCNH2*, and *SCN5A* genes, respectively) being the most common and accounting for approximately 75% of genotype-positive LQTS population^3^.

The *KCNQ1* (Kv7.1) and *KCNH2* (hERG or Kv11.1) genes respectively encode *α*-subunits of slow (*I*_Ks_) and rapid (*I*_Kr_) components of channels mediating delayed rectifier potassium (K^+^) currents. *I*_Ks_ and *I*_Kr_ are two major outward repolarizing K^+^ currents during the plateau and repolarization phases of the cardiac action potential (AP), and play a critical role in controlling the ventricular AP duration (APD)^5,6^. The *SCN5A* gene encodes the *α*-subunit of the predominant cardiac sodium channel (Na_V_1.5) that conducts the depolarizing sodium inward current and is mainly responsible for the initial depolarization in cardiomyocytes. Mutations in *KCNQ1*, *hERG* and *SCN5A* can cause LQTS through either a loss-of-function of potassium channels (*I*_Ks_ and *I*_Kr_) or a gain-of-function of sodium channel leading to an increase in the late *I*_Na_, lengthening the cardiac APD and manifesting as a prolonged QT interval^7^.

About 4–11% of LQTS patients host multiple mutations and typically present at a younger age with a more severe cardiac phenotype compared with individuals carrying a single mutation^8–10^. Patients with compound mutations were found to be associated with longer QTc, more frequent cardiac events, and earlier onset of cardiac events. However, the underlying mechanisms remain unclear.

We identified a LQTS family harboring three compound mutations in different genes: one missense mutation in *KCNQ1* (R174C), one nonsense mutation in *hERG* (E1039X) and another missense mutation in *SCN5A* (E428K). To the best of our knowledge, this is the first report of LQTS associated with three different rare variants. We characterized the functional consequences of the *I*_Ks_, *I*_Kr_ and *I*_Na_ channels reconstituted with these three mutations in mammalian cells and provide important insight into molecular mechanisms underlying the LQTS associated with compound mutations. Specifically, we found that recombinant channel ‘*I*_Ks_’ and ‘*I*_Kr_’ interact and mutations in the two *α* subunits might produce a synergistic lesion in cardiac channel function. These findings may explain why patients with compound mutations show a more severe phenotype than those carrying a single mutation and suggest that the management of such patients should be tailored to their increased risk for arrhythmias^8–11^.

## Results

### Case description

**Figure 1:**
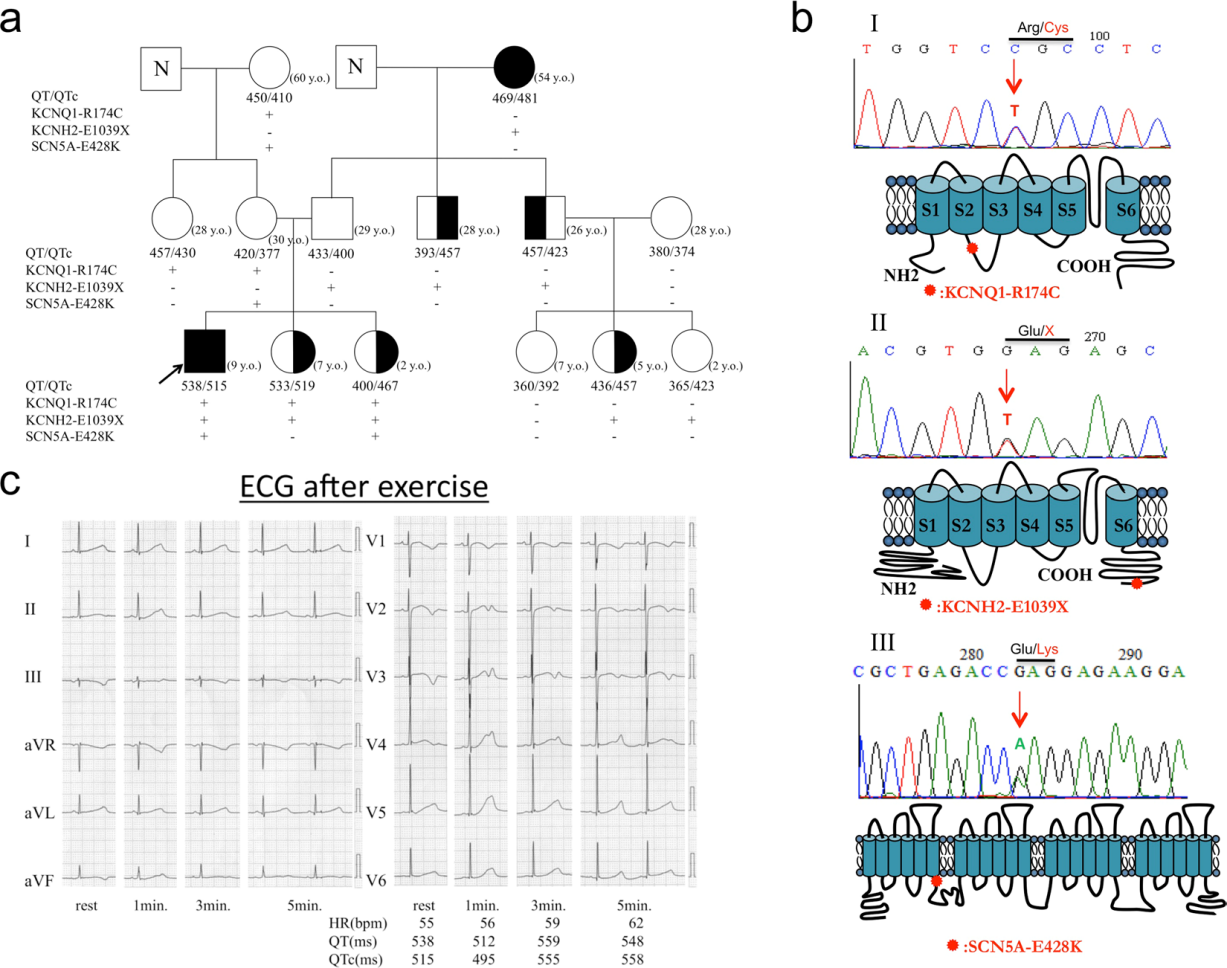
Molecular discovery and clinical characterization of three-mutation carriers. (**a**) The pedigree of three-mutation carriers. Square and circle symbols represent male and female subjects, respectively. Left solid symbols (◧ and ◐) indicate syncope carriers, right solid symbols (◐ and ◧) indicate QTc prolongation, arrow indicates proband, N mark indicates a family member whose information is not available, and QTc = corrected QT interval. (**b**) Representative result of deoxyribonucleic acid sequence analysis and predicted topology of *I*_Ks_ (I), *I*_Kr_ (II), and *I*_Na_ (III) channels. Red arrows and solid circle symbols indicate the location of three mutations in sequence and topology, respectively (**c**) Twelve-lead electrocardiograms of proband at rest and after exercise.

The index patient was a 9-year-old boy (indicated by arrow in family pedigree of Fig. 1a), who experienced repetitive syncope while playing at school. He was identified as carrying three heterozygous mutations in three different genes: p.R174C (c.520 C > T) in *KCNQ1*, p.E1039X (c.3115 G > T) in *hERG*, and p.E428K (c.1282 G > A) in *SCN5A*. Figure 1b shows locations of three mutations in the relevant ion channel protein. The proband was admitted to a nearby hospital and diagnosed with LQTS. The Schwartz score was 4.5 points (T-wave alternans, notched T wave in three leads, low HR for age and syncope with stress). His basal ECG showed a negative T wave in lead III, aVF and V_1_-V_3_, and treadmill stress test uncovered a greater QT prolongation and the appearance of biphasic T wave on exercise (Fig. 1c).

The proband had a family history of syncope and QT prolongation (Fig. 1a). Clinical and genetic analysis of his familial members revealed that his 2-year-old sister also carried all three mutations and another younger sister had both *KCNQ1*-R174C and *hERG*-E1039X mutations. Both sisters showed QT prolongation, but were asymptomatic. All members with one (*KCNQ1*-R174C) or two compound mutations (*KCNQ1*-R174C + *SCN5A*-E428K) in his mother’s family were asymptomatic and showed no QT prolongation. In LQT1 patients, QT prolongation can be sometimes detected along with the increase of heart rate^12^, however, they did not agreed for the exercise stress test. In his father’s family (*hERG*-E1039X carriers), the grandmother (17-year-old at onset time) suffered syncope by a telephone ringing while she was sleeping, which is typical for LQT2. One of the proband’s uncles experienced syncope, and 3/6 mutation carriers showed QT prolongation. Compared with QTc intervals in single *hERG*-E1039X carriers (440.2 ± 12.2 ms, n = 6), those in *hERG*-E1039X carriers with additional mutations (500.3 ± 16.7 ms, n = 3) were significantly longer (*P < *0.05).

## Electrophysiological study

### The KCNQ1-R174C mutation produced a mild inhibitory effect on ‘*I*_Ks_’

**Figure 2:**
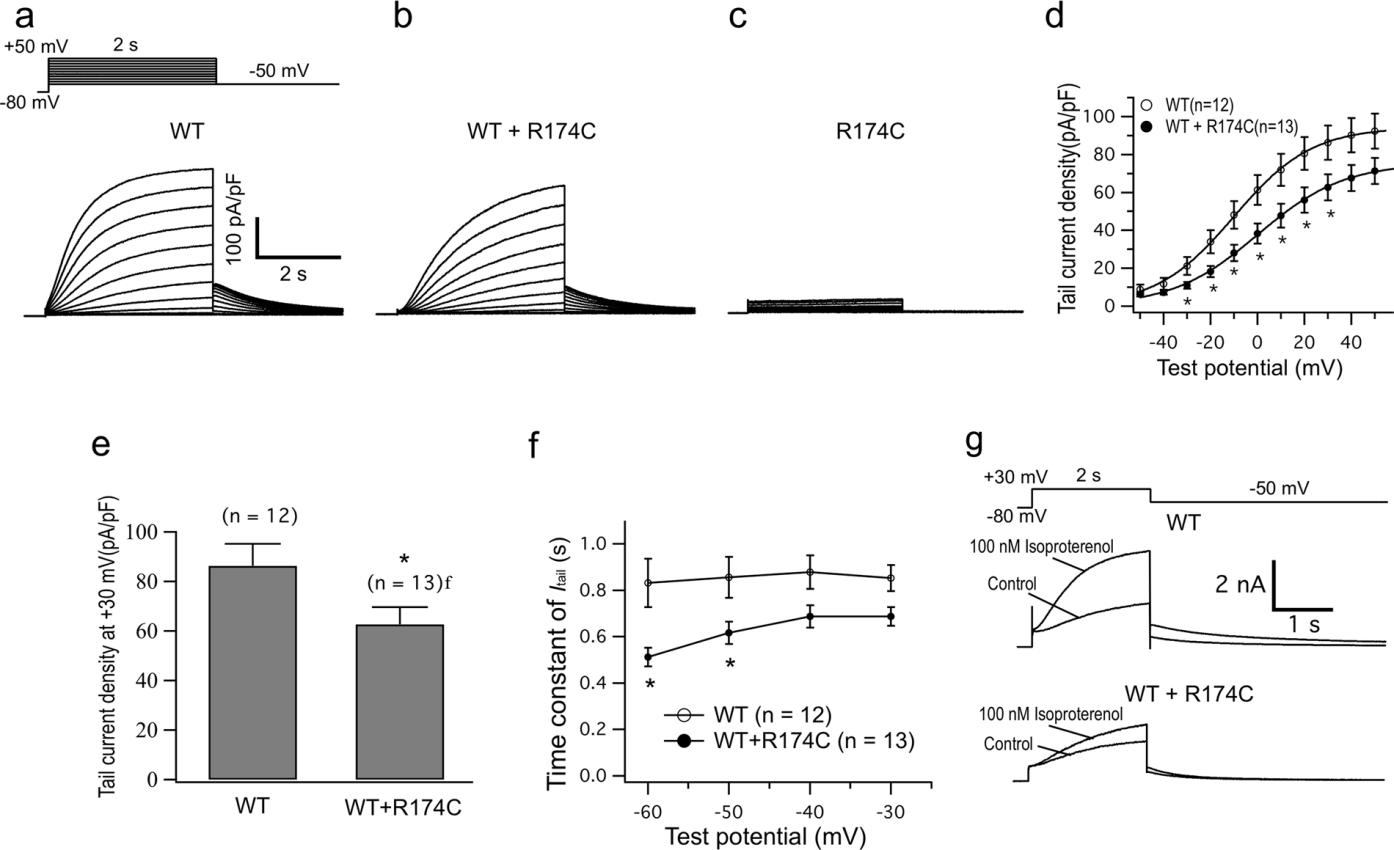
*KCNQ1*-R174C mutation produced a mild inhibitory effect on ‘*I*_Ks_’. Representative current traces recorded from CHO cells expressing KCNE1 with KCNQ1-WT (**a**), KCNQ1-WT + KCNQ1-R174C (**b**) and KCNQ1-R174C (**c**). (**d**) Current-voltage (*I*-*V*) relations for tail *I*_Ks_ elicited after the voltage-step to −50 mV from various test potentials. Protocol is shown as inset to ‘A’. (**e**) Mean peak *I*_Ks_ tail densities recorded on repolarization to −50 mV following 2-s depolarization to +30 mV for the different transfection conditions. (**f**) Deactivation time constants (*τ*) obtained by fitting the tail *I*_Ks_ decay to a single exponential function for voltages between −60 mV and −30 mV. (**g**) Superimposition of *I*_Ks_ traces recorded from HEK 293 cells expressing Yotiao + KCNE1 with KCNQ1-WT or KCNQ1-WT + KCNQ1-R174C before and after bath application of 100 nmol/L isoproterenol. *P < 0.05 *vs* WT.

Figure 2a–c show representative whole-cell current traces recorded from CHO cells expressing KCNE1 with KCNQ1-WT, KCNQ1-WT + KCNQ1-R174C and KCNQ1-R174C, respectively. Both the steady state and tail *I*_Ks_ amplitude in WT + R174C conditions were mildly decreased compared to WT alone, whilst R174C KCNQ1 alone produced no currents. Figure 2d shows the current-voltage (*I* - *V*) relations for *I*_Ks_ tails elicited after the voltage-step to −50 mV from various test potentials. Figure 2e summarizes *I*_Ks_ tail densities measured at +30 mV. Compared with WT, WT +R174C KCNQ1 significantly decreased *I*_Ks_ densities for voltages between −30 mV and +30 mV (Fig. 2d,e and Table 1). Voltage-dependent activation was quantified by fitting a Boltzmann equation to the *I*-*V* relations, and resultant data show that WT + R174C KCNQ1 significantly increased the *V*_h_ value (Table 1).

**Table 1:** Biophysical kinetics of KCNQ1/KCNE1 channels in CHO cells.

Parameters	n	Tail current density at 30 mV (pA/pF)	Activation gate		τ(s) of deactivation at	
			*V*_h_ (mV)	*k* (mV)	−50 mV	−60 mV
Q1-WT	12	86.31 ± 8.94	−9.14 ± 2.41	15.78 ± 0.51	0.86 ± 0.09	0.83 ± 0.10
Q1-WT + Q1-R174C	15	62.70 ± 6.88*	2.89 ± 2.83**	18.89 ± 1.58	0.77 ± 0.06*	0.64 ± 0.05**
Q1-R174C	6	0	—	—	—	—
Q1-WT + hERG-WT	10	132.30 ± 12.82**	−15.71 ± 5.40	19.00 ± 1.48*	1.70 ± 0.30*	2.29 ± 0.45*
Q1-R174C + hERG-WT	6	0	—	—	—	—
Q1-WT + hERG-E1039X	11	146.88 ± 16.31**	−9.65 ± 2.62	18.89 ± 1.27*	1.07 ± 0.09^#^	1.19 ± 0.18^#^
Q1-R174C + hERG-E1039X	5	0	—	—	—	—

Data are mean ± SEM. n = number of tested cells; Q1 = KCNQ1; *V*_h_ and *k* = midpoint potential and slope factors, respectively. *P < 0.05 *vs* Q1-WT, **P < 0.01 *vs* Q1-WT; ^#^*P* < 0.05 *vs* Q1-WT + hERG^-^WT.

Deactivation rates for *I*_Ks_ were measured by depolarizing cells to +30 mV for 2 s, followed by repolarizing steps from −60 mV to −30 mV in 10-mV increments. Figure 2f shows the time constant for deactivation plotted as a function of repolarization potential. Compared with WT, WT + R174C significantly accelerated the deactivation rates between −60 mV and −50 mV (Table 1). Overall, the R174C mutation exerted a mild inhibitory effect on KCNQ1-WT channel^13^.

As the proband experienced syncopal episodes while playing with his classmates and his QTc interval was prolonged by exercise (Fig. 1c), we further tested whether *KCNQ1-*R174C might impair the response of *I*_Ks_ to adrenergic stimulation in HEK293 cells co-expressing WT + R174C KCNQ1 with KCNE1 and Yotiao^12^. As typically shown in Fig. 2g, 100 nM isoproterenol increased *I*_Ks_ by 93.5 ± 15.8% (n = 18) in cells expressing WT alone, but only mildly increased *I*_Ks_ by 50 .4 ± 7.6%, (n = 13, *P < *0.05 *vs* WT) in cells expressing WT + R174C KCNQ1. The result suggests that WT + R174C KCNQ1 partially blunted the activation of *I*_Ks_ in response to isoproterenol, which is consistent with a previous report on the response of this mutant channel to forskolin in *Xenopus laevis* oocytes^13^.

### HERG-E1039X mutation caused an incomplete loss-of-function in ‘*I*_Kr_’

**Figure 3:**
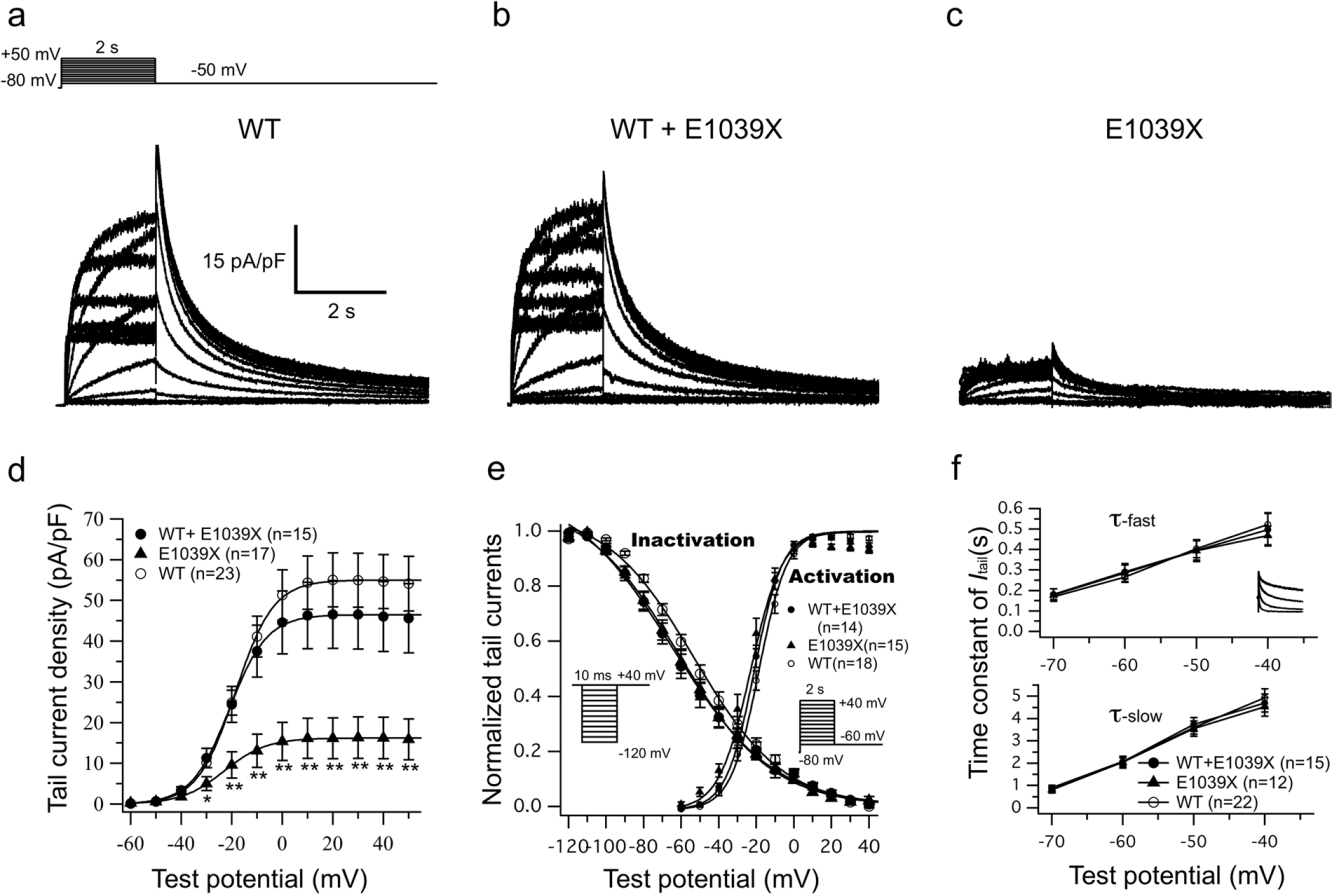
*HERG*-E1039X mutation caused an incomplete loss-of-function in ‘*I*_Kr_’. Representative current traces recorded from CHO cells expressing hERG-WT (**a**), hERG-WT + hERG-E1039X (**b**) and hERG-E1039X (**c**), respectively. Protocol shown as inset to ‘A’. (**d**) *I*-*V* relations for tail *I*_Kr_ elicited after the voltage-step to −50 mV from various test potentials. *P < 0.05 *vs* WT, **P < 0.01 *vs* WT. (**e**) Normalized activation and steady-state inactivation were determined by means of the voltage protocols shown in the inset. (**f**) Deactivation time constants (τ) obtained by fitting the tail *I*_Kr_ decay (the inset in the upper panel) to a bi-exponential function for voltages between −70 mV and −40 mV.

Figure 3a–c show representative whole-cell current traces recorded from CHO cells expressing hERG-WT, hERG-WT + hERG-E1039X and hERG-E1039X, respectively. *I*-*V* relations in Fig. 3d indicates that, although the steady state and tail *I*_Kr_ amplitudes in E1039X hERG alone were notably decreased, the *I*_Kr_ amplitudes in WT + E1039X hERG were not significantly changed (Table 2).

**Table 2:** Biophysical kinetics of hERG channels in CHO cells.

Parameters	Tail current density at +50 mV (pA/pF)	Activation gate		Inactivation gate		τ of recovery from inactivation at −40 mV (ms)	Deactivation at −40 mV	
		*V*_h_ (mV)	*k* (mV)	*V*_h_ (mV)	*k* (mV)		τ-f (s)	τ-f (s)
hERG-WT	54.09 ± 6.67(25)	−18.68 ± 1.27	7.17 ± 0.35(23)	−52.50 ± 3.04	−22.05 ± 0.59(18)	2.90 ± 0.20(18)	0.52 ± 0.06	4.92 ± 0.40(22)
hERG-WT + hERG-E1039X	45.64 ± 8.51(18)	−22.06 ± 1.29	6.67 ± 0.27(17)	−64.62 ± 3.75*	−23.98 ± 1.15(14)	3.70 ± 0.35(14)*	0.47 ± 0.05	4.53 ± 0.43(9)
hERG-E1039X	15.86 ± 5.05(36)**	−22.99 ± 2.03	7.05 ± 0.40(15)	−64.63 ± 4.07*	−23.28 ± 1.02(14)	3.85 ± 0.39(14)*	0.50 ± 0.08	4.69 ± 0.41(9)
hERG-WT + Q1-WT	32.75 ± 4.96(21)*	−7.97 ± 2.04**	8.55 ± 0.23(24)*	−67.71 ± 3.08**	−30.30 ± 0.85(16)**	9.70 ± 1.20(10)**	0.52 ± 0.03	5.81 ± 0.39(24)
hERG-E1039X + Q1-WT	16.63 ± 4.82(21)**†	−12.15 ± 2.08**	9.19 ± 0.91(15)*	−64.97 ± 3.41**	−29.55 ± 1.44 (9)**	12.62 ± 3.18(7)**	0.54 ± 0.07	5.45 ± 0.07(11)
hERG-WT + Q1-R174C	32.82 ± 5.75(12)*	−8.90 ± 2.00**	7.92 ± 0.46(12)	−66.41 ± 4.00*	−30.42 ± 1.85 (8)**	8.16 ± 1.14(8)**	0.55 ± 0.07	5.60 ± 0.07(10)
hERG-E1039X + Q1-R174C	15.41 ± 4.06(17)**^##^	−8.52 ± 3.16**	8.49 ± 0.50(15)*	−65.89 ± 3.20**	−31.58 ± 1.70(11)**	10.11 ± 2.15(6)**	0.57 ± 0.05	5.89 ± 0.07(9)

Data are mean ± SEM. Numbers in parentheses = tested cells; Q1 = KCNQ1; *V*_h_ and *k* = midpoint potential and slope factors, respectively;

τ_f_ and τ_s_ = fast and slow deactivating time constant, respectively. *P < 0.05 *vs* hERG-WT, **P < 0.01 *vs* hERG-WT;

^†^*P* < 0.05 *vs* hERG-WT + Q1-WT; ^##^*P* < 0.05 *vs* hERG-WT + Q-R174C.

Figure 3e and f show normalized voltage dependence of activation/inactivation curves and time constants for deactivation under three different conditions, respectively. Numerical data pertaining to the biophysical properties therein are summarized in Table 2. Compared with those of WT hERG (*V*_h_: −52.50 ± 3.04 mV; *τ* of recovery from inactivation at −40 mV: 2.90 ± 0.20 ms; n = 18), however, the *V*_h_ for the steady state inactivation of both E1039X (−64.63 ± 4.07 mV, n = 14) and WT + E1039X (−64.62 ± 3.75 mV, n = 14) showed a marked (*P < *0.05 *vs* WT) negative shift, and the time course (*τ*) of recovery from inactivation at −40 mV was significantly (*P < *0.05 *vs* WT) slower for both E1039X (3.85 ± 0.39 ms, n = 14) and WT + E1039X (3.70 ± 0.35 ms, n = 14). The pronounced hyperpolarizing shift of inactivation and slowed recovery from inactivation are likely to decrease the *I*_Kr_ channel availability during excitation and to cause an incomplete loss-of-function in *I*_Kr_. The parameters for activation and time constants for deactivation were not significantly different between WT, E1039X and WT + E1039X hERG (Table 2).

### SCN5A-E428K increased the peak ‘*I*_Na_’ currents but produced no late ‘*I*_Na_’

**Figure 4:**
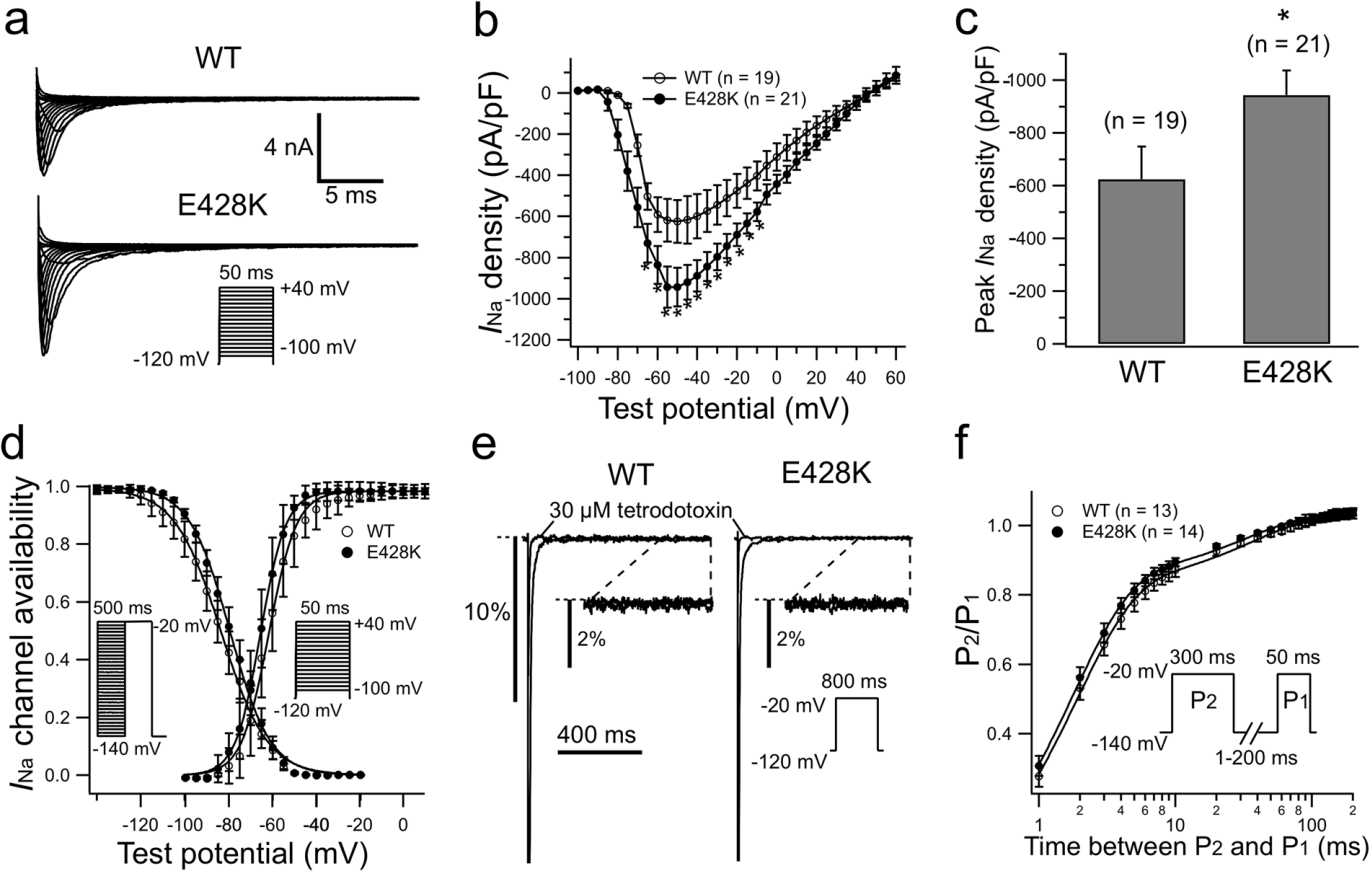
*SCN5A*-E428K mutation decreased the peak currents in ‘*I*_Na_’. (**a**) Representative current traces recorded from CHO cells expressing h*β*_1_ with SCN5A-WT or SCN5A-E428K. Inset shows voltage protocol. (**b**) *I*-*V* relations for peak *I*_Na_ densities of WT and E428K channels. (**c**) Mean peak *I*_Na_ densities of WT (at −50 mV) and E428K (at −55 mV) channels. (**d**) Voltage dependence of relative *I*_Na_ conductance activation and steady-state inactivation were determined by means of the voltage protocols shown in the inset. (**e**) Representative late *I*_Na_ currents recorded in the absence or presence of 30 μM TTX. (**f**) The time course of recovery from inactivation of *I*_Na_ was elicited with a double pulse protocol. *P < 0.05 *vs* WT.

Figure 4a shows representative whole-cell current traces recorded from CHO cells expressing h*β*_1_ with SCN5A-WT or SCN5A-E428K. Figure 4b shows *I*-*V* relations for peak *I*_Na_ elicited by the protocol shown in the inset, and Fig. 4c summarizes peak *I*_Na_ densities. Compared with WT, E428K SCN5A significantly increased peak *I*_Na_ densities between −70 mV and −10 mV. The peak *I*_Na_ density of E428K was 943.9 ± 93.8 pA/pF (n = 21) at −55 mV, which is significantly (*P* < 0.05) larger than that of WT (625.4 ± 124.4 pA/pF, n = 19,) at −50 mV (Fig. 4c). Figure 4d and f show conductance-voltage and steady state inactivation curves, representative late *I*_Na_ traces recorded in the presence of 30 μM tetrodotoxin (TTX), and the properties of *I*_Na_ recovery from inactivation for WT and E428K SCN5A. The mutation caused no significant changes to these parameters.

### Co-expression of KCNQ1/KCNE1 with hERG

Based on the above electrophysiological findings on the three mutations, two loss-of-function mutations of potassium channels appeared to cause the clinical phenotype in this relatively large LQTS family. The proband’s two sisters carrying the same combination of heterozygous compound *KCNQ1* and *hERG* mutations showed long QT features very similar to those of the proband, suggesting that the pathogenesis of triple mutation carriers was mainly associated with the *KCNQ1*-R174C and *hERG*-E1039X. We therefore examined the interaction between KCNQ1-R174C and hERG-E1039X by co-expressing the two mutations into CHO cells.

**Figure 5:**
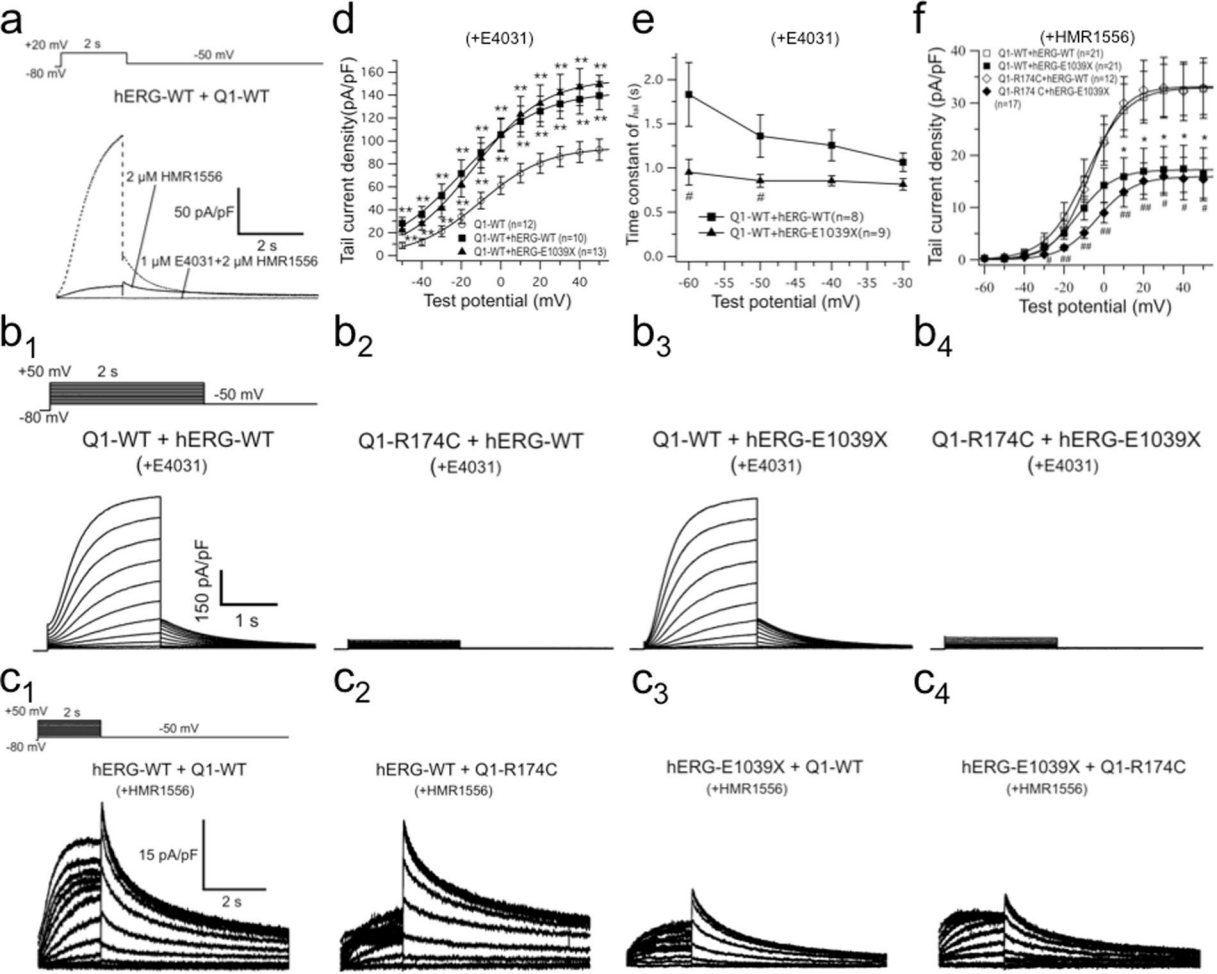
Interaction between ‘*I*_Ks_’ and ‘*I*_Kr_’ when KCNQ1/KCNE1 and hERG were co-expressed together. (**a**) Representative current traces recorded from CHO cells expressing with hERG-WT + KCNQ1-WT/*KCNE1* in the presence of 2 μM HMR1556 and 1 μM E4031. (**b**) and (**c**) show representative current traces recorded from CHO cells expressing KCNQ1-WT/KCNE1 + hERG-WT, KCNQ1-R174C/KCNE1 + hERG-WT, KCNQ1-WT/KCNE1 + hERG-E1039X, and KCNQ1-R174C/KCNE1 + hERG-E1039X, in the presence of 1 μM E4031 (**b**_**1**_–**b**_**4**_) or 2 μM HMR1556 (**c**_**1**_–**c**_**4**_). Protocols shown as insets to ‘B_1_’ and ‘C_1_’, respectively. (**d**) *I*-*V* relations for tail currents elicited after the voltage-step to −50 mV from various test potentials in the presence of E4031 (except for KCNQ1-WT). **P < 0.01 *vs* KCNQ1-WT. (**e**) Deactivation time constants (*τ*) obtained by the same method as Fig. 2f in the presence of E4031. ^#^*P* < 0.05 *vs* KCNQ1-WT + hERG-WT. (**f**) *I*-*V* relations for tail currents elicited after the voltage-step to −50 mV from various test potentials in the presence of 2 μM HMR1556. *P < 0.05 *vs* hERG-WT + KCNQ1-WT/KCNE1; ^#^*P* < 0.05 *vs* hERG-WT + KCNQ1-WT/KCNE1, ^##^*P* < 0.01 *vs* hERG-WT + KCNQ1-WT/KCNE1.

Figure 5a shows representative current traces recorded from a cell co-expressing KCNQ1-WT/KCNE1 and hERG-WT. In the presence of 1 μM E4031 (Kv11.1/*I*_Kr_ inhibitor) and 2 μM HMR1556 (Kv7.1/*I*_Ks_ inhibitor), the current was totally blocked, which confirms that the current was exclusively composed of KCNQ1 + KCNE1 and hERG channel currents. Figure 5b_1_–b_4_ and c_1_–c_4_ show the representative whole-cell current traces recorded from CHO cells expressing KCNE1 + KCNQ1-WT/KCNQ1-R174C with hERG-WT/ hERG-E1039X in the presence of 1 μM E4031 or 2 μM HMR1556, respectively.

Figure 5d shows *I*-*V* relations for tail currents elicited using the protocol in inset of Fig. 5b_1_ in the presence of 1 μM E4031. Open circles indicate those measured for KCNQ1-WT alone. Compared with KCNQ1-WT, both KCNQ1-WT + hERG-WT (solid squares) and *KCNQ1*-WT + hERG-E1039X (solid triangles) significantly increased the tail current amplitudes (Fig. 5d and Table 1). In addition, KCNQ1-WT + hERG-WT caused a significant negative shift of both *V*_h_ and *K* values of activation curve, and deactivation rates of KCNQ1-WT + hERG-WT were significantly slower than those of KCNQ1-WT between −60 mV and −50 mV (Table 1). These data suggest that hERG exerted a gain-of-function effect on *I*_Ks_ when co-expressed with KCNQ1.

On the other hand, Fig. 5e shows that KCNQ1-WT + hERG-E1039X (solid triangles) significantly accelerated deactivation times between −60 mV and −50 mV (Table 1) compared with KCNQ1-WT + hERG-WT (solid squares), which suggests that *hERG*-E1039X led to altered *I*_Ks_ kinetics when co-expressed with KCNQ1, although tail current densities were not significantly affected (Table 1).

Figure 5f shows *I*-*V* relations for tail currents elicited using the protocol in inset of Fig. 5c_1_ in the presence of 2 μM HMR1556. Compared with those of hERG-WT, tail currents and the *V*_h_ values for the steady state inactivation of both hERG-WT + KCNQ1-WT and hERG-WT + KCNQ1*-*R174C were significantly lower, but the *V*_h_ values for voltage-dependent activation and the recovery time from inactivation of both hERG-WT + KCNQ1-WT and hERG-WT + KCNQ1*-*R174C were significantly higher at −40 mV (Table 2). Taken together, KCNQ1 (including KCNQ1*-*R174C mutant channels) attenuated *I*_Kr_ when co-expressed with hERG. In the meantime, Fig. 5f and Table 2 show that the tail current of hERG-E1039X + KCNQ1*-*WT (solid squares) was lower than that of hERG-WT + KCNQ1-WT (open squares) and the tail current of hERG-E1039X + KCNQ1*-*R174C (solid diamonds) was lower than that of hERG-WT + KCNQ1-R174C (open diamonds), which supports the above data that hERG-E1039X caused a loss-of-function in *I*_Kr_ even in the condition of co-expression with KCNQ1. However, we failed to detect any significant changes in parameters between hERG-WT + KCNQ1-WT and hERG-WT + KCNQ1-R174C or between hERG-E1039X + KCNQ1-WT and hERG-E1039X + KCNQ1-R174C, which implicates that KCNQ1-R174C did not affect the function of *I*_Kr_ when co-expressed with hERG.

### Expression of channel tetramers on cell membrane was disrupted by either KCNQ1-R174C or hERG-E1039X but rescued by co-expression of WT

**Figure 6:**
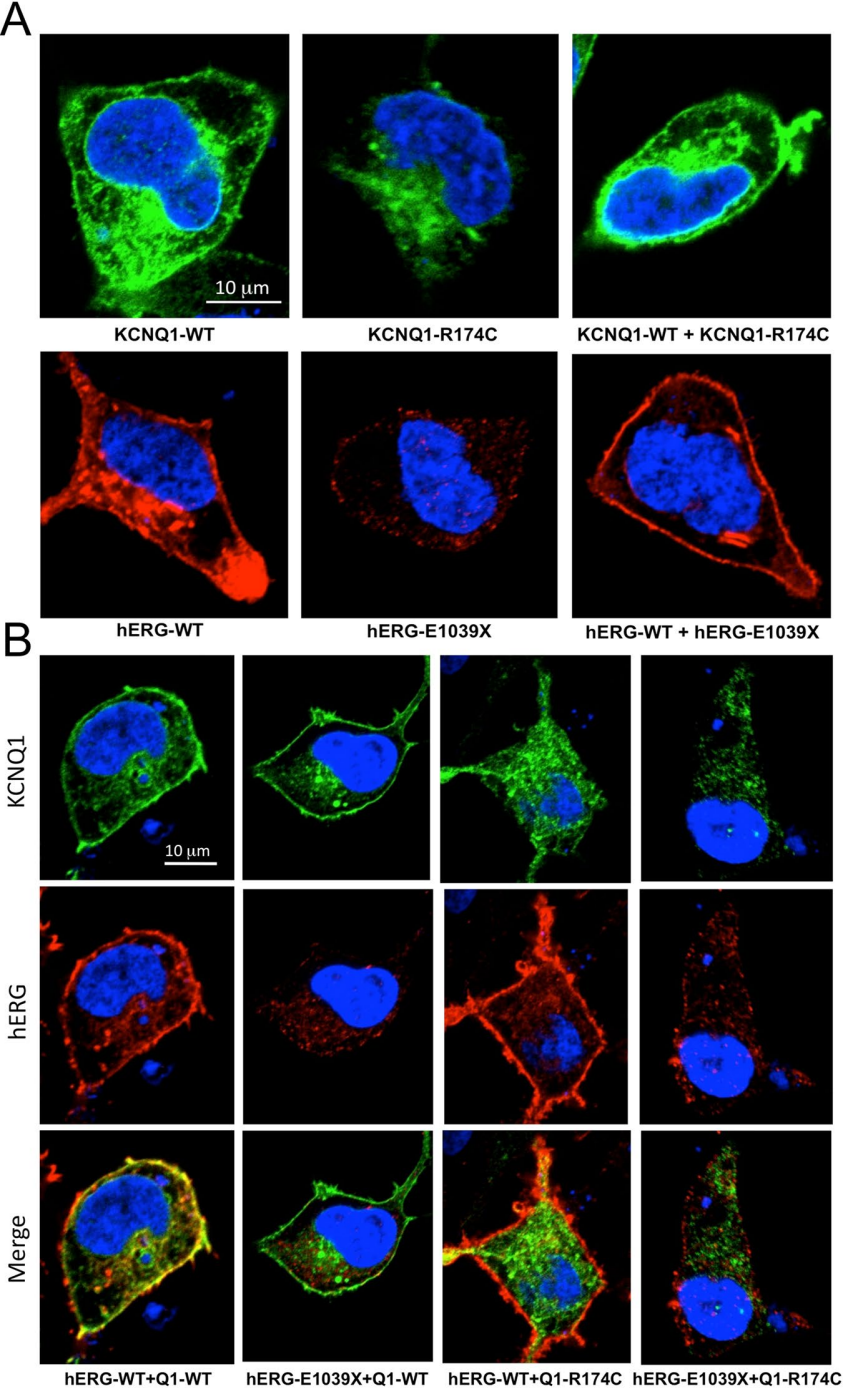
Cellular localization of KCNQ1 and hERG proteins in CHO cells. Confocal images of upper panels in (**A**) are shown of KCNQ1 (labelled by green) in representative CHO cells co-expressing KCNE1 with KCNQ1-WT, KCNQ1-WT + KCNQ1-R174C, and KCNQ1-R174C, respectively. The lower panels in (**a**) are confocal images of hERG (labelled by red) in representative CHO cells co-expressing hERG-WT, hERG-WT + hERG-E1039X and hERG-E1039X, respectively. (**B**) Confocal images of KCNQ1 and hERG proteins in CHO cells co-expressing KCNQ1-WT*/*KCNE1 + hERG-WT, KCNQ1-WT/KCNE1 + hERG-E1039X, KCNQ1-R174C*/*KCNE1 + hERG-WT, and KCNQ1-R174C*/*KCNE1 + hERG-E1039X, respectively. Upper panels in (**b**) are images of KCNQ1 (labelled by green), middle panels are images of hERG (labelled by red), and lower panels are merged images of KCNQ1 + hERG (labelled by yellow). Cells were immunostained with an anti-KCNQ1 or an anti-hERG antibodies. Nuclei were immunostained with DAPI.

Figure 6A shows confocal images of CHO cells expressing KCNQ1/KCNE1 (upper panels) and hERG (lower panels). Both of KCNQ1-WT and hERG-WT proteins were amply transported to the cell membrane. In cells expressing KCNQ1-R174C alone, mutant proteins were mostly distributed in the cytosol but scarcely on the cell membrane, suggesting the trafficking defect of channel protein. While the hERG-E1039X mutant proteins were less presented both in cytosol and on cell membrane, implicating the inhibited protein synthesis and/or its potentiated degradation by hERG-E1039X. However, in cells expressing KCNQ1-WT + KCNQ1-R174C or hERG-WT + hERG-E1039X, channel proteins were expressed both on the cell membrane and in the cytosol, suggesting that the cell membrane expression of channel proteins was increased by the co-expression of WT subunits.

Figure 6B shows confocal images of CHO cells co-expressing (from left to right) KCNQ1-WT/KCNE1 + hERG-WT, KCNQ1-WT/KCNE1 + hERG-E1039X, KCNQ1-R174C/KCNE1 + hERG-WT, and KCNQ1-R174C/KCNE1 + hERG-E1039X. The upper (or middle) panels show that in cells co-expressing KCNQ1-WT/KCNE1 + hERG-WT and KCNQ1-WT/KCNE1 + hERG-E1039X (or middle panels: KCNQ1-R174C/KCNE1 + hERG-WT), KCNQ1 (or middle panels: hERG) proteins were expressed both on the cell membrane and in the cytosol. But, in cells (upper panels) co-expressing KCNQ1-R174C/KCNE1 + hERG-WT (or -E1039X), KCNQ1-R174C proteins were mostly distributed in the cytosol. On the other hand, in cells (middle panels) expressing hERG-E1039X + KCNQ1-WT (or -R174C)/KCNE1, hERG-E1039X proteins were less present both in cytosol and on cell membrane. These data are consistent with those in Fig. 6A, and suggest that KCNQ1 and hERG did not affect the expression pattern of proteins one-another, which is also confirmed by the merged figures in lower panels.

## Discussion

Although LQTS caused by two compound mutations is relatively common, the arrhythmia associated with three different compound mutations is a rare case, which accounts for 0.2% of our LQTS cohort. The present study on the three-compound mutation case indicated that *KCNQ1*-R174C produced a mild inhibitory effect on *I*_Ks_ and *hERG*-E1039X caused an incomplete loss-of-function in *I*_Kr_. In addition, the present study showed that *I*_Ks_ and *I*_Kr_ interact: it is striking that *hERG*-E1039X caused a loss-of-function in *I*_Ks_ when co-expressed with KCNQ1, which is likely to exacerbate the dysfunction of *I*_Ks_ caused by *KCNQ1*-R174C. This result might reveal why compound mutations are associated with increased arrhythmic risk.

Of the three pathogenic mutations in the present study, *KCNQ1*-R174C was previously reported to be associated with both heterozygous LQT1^14^ and homozygous autosomal-recessive LQT1^15^, in which the homozygous *KCNQ1*-R174C carrier displayed extreme QT prolongation and suffered multiple breakthrough cardiac events before succumbing to his malignant LQTS phenotype. Our clinical data show that patients with heterozygous *KCNQ1*-R174C or *KCNQ1*-R174C/*SCN5A*-E428K compound mutation were asymptomatic and their QTc intervals were not prolonged (Fig. 1a), suggesting that the phenotype caused by heterozygous *KCNQ1*-R174C is not severe and individuals carrying the same mutation exhibit diverse cardiac phenotypes clinically^13–15^. These results are well explained with our electrophysiological data: KCNQ1-WT + KCNQ1-R174C produced a mild inhibitory effect on *I*_Ks_ channel and KCNQ1-R174 alone produced no *I*_Ks_ current (Fig. 2b–d and Table 1). Immunocytochemical study show that, same as the cell surface expression pattern of another *KCNQ1* mutation G269S^12^, the trafficking-deficiency in the homologous KCNQ1-R174C channel was greatly rescued by co-expression with the WT subunit (upper panels in Fig. 6A), resulting in the increased expression of channel proteins on the cell membrane. This result further explains clinical phenotypes of *KCNQ1*-R174C mutation carriers. Consistent with a previous finding that *KCNQ1*-R174C blunted the increase in *I*_Ks_ with forskolin in *Xenopus laevis* oocytes^13^, we found that *KCNQ1*-R174C blunted the increase in *I*_Ks_ with isoproterenol, which further confirms our previous speculation: a patient with *KCNQ1* mutation showing an excessive prolongation of QT intervals on exercise is likely due to an adrenergic up-regulation of *I*_Ca,L_ without concomitant up-regulation of *I*_Ks_^12^.

*SCN5A*-E428K was reported to be linked to atrial fibrillation (AF)^16,17^. The present clinical data show that two mutation carriers (harboring *KCNQ1*-R174C simultaneously) in proband’s mother family were asymptomatic and did not exhibit QTc interval prolongation (Fig. 1a). Electrophysiological study revealed that SCN5A-E428K increased peak *I*_Na_ but did not affect the late *I*_Na_, which indicates that this mutation might be associated with such genetic disorders as AF rather than LQTS because the increase of the late *I*_Na_ is a characteristic indicator for LQT3^18,19^. In addition to above data, we predicted the pathogenicity of substitutions in *SCN5A*-E428K and *KCNQ1*-R174C mutations through the PolyPhen-2 system^20^. The results show that *SCN5A*-E428K is relatively benign, whereas the *KCNQ1*-R174C is strongly considered to be damaging.

*HERG*-E1039X is a novel nonsense mutation in distal C-terminus. Clinical data show that two of *hERG*-E1039X mutation carriers experienced syncope and half of the mutation carriers showed QT prolongation in proband’s father family (Fig. 1a). Functional analysis indicates that *hERG*-E1039X mutation shifted the inactivation curve of *I*_Kr_ in the hyperpolarizing direction and decelerated the time of recovery from inactivation (Table 2), while other gating kinetics and the current density were not significantly affected. Previous studies postulated that nonsense mutations in *hERG* cause abnormal transcription/translation of *I*_Kr_^21^. The present immunocytochemical data show that, in cells expressing hERG*-*WT + hERG*-*E1039X, channel proteins were amply expressed both on the cell membrane and in the cytosol (lower panels in Fig. 6A), suggesting that channel protein expression in heterozygous channels was very similar to that in WT channels although E1039X mutant alone disrupted the biosynthesis and/or degradation of hERG channel protein. The distinguishing features of *I*_Kr_ kinetics are the rapid voltage-dependent inactivation and recovery from inactivation, subsequently coupling with a slow deactivation^21,22^. Most LQT2-causing mutations associated with abnormal channel gating/kinetics are involved in the accelerated deactivation^21,23,24^. Only a few studies reported that the loss-of-function in *I*_Kr_ caused by hERG channel pore missense mutations (V644L^24^, G584S^25^, V603L and A614V^26^) was associated with channel inactivation. The present study provides evidence that E1039X, a nonsense mutation located in hERG’s distal C-terminus, caused LQT2 through inactivation mechanism, which gives us two notions: (1) in addition to affecting gene transcription/translation, a nonsense mutation in *hERG* can lead to LQT2 through disrupting inactivation gating of *I*_Kr_; (2) the distal C-terminus is also involved in the inactivation in *I*_Kr_. In the present study, we cannot rule out the possibility that a nonsense-mediated mRNA decay (NMD) is involved in the phenotype of patients carrying *hERG*-E1039X. The position of E1039X is close to the other two *hERG* nonsense mutations (W1001 × and R1014X) which were reported to degrade mutant mRNA by NMD and to be associated with LQT2^21^.

The functional interaction between *I*_Kr_ and *I*_Ks_ is still in dispute^27–29^. Ren *et al*. showed that transiently expressed WT or mutant *KCNQ1* downregulated Kv11.1 in both CHO and HEK 293 cells stably expressing hERG and the interactions of two channels occurred via the C terminus^30^, whose findings are consistent with the present study: both *KCNQ1*-WT and *KCNQ1-*R174C mutant decreased hERG channel currents when co-expressed with hERG in CHO cells. When co-expressed with KCNQ1, C-terminal mutation hERG-E1039X disrupted KCNQ1/KCNE1 currents, whist hERG-WT produced a different effect in KCNQ1/KCNE1 currents. These data also support the above study that hERG C-terminus is involved in the interaction between *I*_Kr_ and *I*_Ks_^30^.

It is well known that compound mutation carriers exhibit a more severe phenotype than those with a single mutation^8–11^. Westenskow at al found that a *KCNQ1* mutant and a *KCNE1* mutant could lead to cumulative lesion in *I*_Ks_^8^. Biliczki *et al*. reported that *I*_Ks_ blocker chromanol 293B alone did not markedly lengthen dog ventricular APD, however, when repolarization had already been prolonged by *I*_Kr_ blocker dofetilide, inhibition of *I*_Ks_ with same concentration of chromanol 293B substantially delayed repolarization^31^. Their data suggest a synergistic prolongation of repolarization produced by *I*_Kr_ and *I*_Ks_ blockade. In the present experiment, *hERG*-E1039X caused a loss-of-function in KCNQ1/KCNE1 channels. This result indicates that a mutation in *hERG* not only can disrupt *I*_Kr_ but can worsen *I*_Ks_ function and superimpose to cause a synergistic lesion to the defective *I*_Ks_ encoded with a mutant *KCNQ1*, leading to further prolongation of APD and the QT interval. Our *in silico* study also confirms that the synergistic effects of *KCNQ1*-R174C and *hERG*-E1039X in *I*_Ks_ could prolong APD markedly (see Supplementary Material). Therefore, although phenotype of heterozygous *KCNQ1*-R174C carriers are mild, patients harboring additive pathogenic mutation *hERG*-E1039X showed more severe QT interval prolongations because of superimposed *I*_Ks_ lesion caused by *hERG*-E1039X mutation and the proband even experienced a syncope evoked by exercise^32^. Based on the above functional consequence, we suggest that compound pathogenic mutation carriers should be tailored to their increased risks for arrhythmias because these patients are more readily to be predispose to fatal arrhythmias. For example, a *hERG*-*KCNQ1* compound mutation carrier should avoid QT-prolonging medications and swimming.

## Conclusion

We identified a LQTS family harboring three pathogenic mutations in different genes and characterized the functional consequences of related three mutant channels. The synergistic lesion caused by different pathogenic mutation is very likely why patients with compound mutations showed a relatively more severe phenotype.

## Methods

### Clinical investigation and genetic testing

The study population consisted of 1,015 consecutive LQTS probands whose diagnosis was referred to the criteria of Schwartz *et al*.^1^. The protocol for genetic analysis was approved by the Institutional Ethics Committee of Shiga University of Medical Science and performed under its guidelines. Written informed consent was obtained from every subject before analysis, in accordance with the last version of the Declaration of Helsinki and with recommendations by the local ethics committee. Genomic deoxyribonucleic acid (DNA) used for genetic evaluation was isolated from venous blood lymphocytes. Genetic screening for mutations in LQTS-related genes including *KCNQ*1, *hERG*, *SCN5A*, *KCNE*1, *KCNE*2, *KCNJ*2 and *CACNA1C* was conducted by denaturing high-performance liquid chromatography (WAVE system, Transgenomic Inc., Omaha, Nebraska). For abnormal screening patterns, sequencing was performed with an automated sequencer (ABI PRISM 3100×, Applied Biosystems, Foster City, California).

### Heterologous expression of cDNA in CHO cells

Full-length complementary deoxyribonucleic acid (cDNA) encoding human wild-type (WT) *KCNQ*1 (GenBank AF000571, kind gift from Dr. J. Barhanin, Institut de Pharmacologie Moleculaire et Cellulaire, CNRS, Valbonne, France) was subcloned into a pIRES2-EGFP expression vector. Full-length cDNA encoding human *KCNE1* (GenBank M26685) subcloned into the pcDNA3.1 expression vector was obtained by polymerase chain reaction from human heart cDNA library (Clontech Laboratories, Mountain View, CA, USA). Full-length cDNA encoding human WT-*hERG* (GenBank AF363636, kind gift from Dr. M. Sanguinetti, University of Utah, Salt Lake City, UT, USA) was subcloned into pRc/CMV expression vector. Full-length cDNA encoding human WT-*SCN5A* (GenBank AB158469) was subcloned into pcDNA3.1 expression vector. Full-length cDNA encoding human sodium channel *β*_1_ subunit (h*β*_1_) was subcloned into a bicistronic plasmid (pEGFP-IRES). *KCNQ1*-R174C, *hERG*-E1039X and *SCN5A*-E428K mutants were constructed using a Quick Change II XL site-directed mutagenesis kit according to the manufacturer’s instructions (Stratagene, La Jolla, California), and they were also subcloned into the pIRES2-EGFP, pRc/CMV and pcDNA3.1 expression vectors, respectively. All mutants were fully sequenced to ensure the fidelity. To constitute *I*_Ks_, *I*_Kr_ or *I*_Na_ channels, *KCNQ1*-WT and/or *KCNQ1*-R174C + *KCNE1* cDNAs (0.5 µg WT and/or 0.5 µg R174C + 0.5 µg *KCNE1*), *hERG*-WT and/or *hERG*-E1039X + green fluorescent protein (GFP) cDNAs (1 µg WT and/or 1 µg E1039X + 0.5 µg GFP), or *SCN5A*-WT and/or *SCN5A*-E428K + h*β*_1_ cDNAs (1.0 µg WT and/or 1.0 µg E428K + 1.0 µg h*β*_1_) were transiently transfected into CHO cells using Lipofectamine (Invitrogen Life Technologies, Inc. Carlsbad, CA, USA) according to the manufacturer’s instructions. In one set of experiments, we also co-transfected *KCNQ*1, *KCNE*1 and Yotiao cDNAs (0.5 µg WT and/or 0.5 µg R174C + 0.5 µg *KCNE1* + 2 µg Yotiao) into human embryonic kidney 293 (HEK293).

### Solutions and chemicals

The pipette solution for potassium channel current recording contained (in mM): 70 potassium aspartate, 40 KCl, 10 KH_2_PO_4_, 1 MgSO_4_, 3 Na_2_-ATP, 0.1 Li_2_-GTP, 5 EGTA and 5 HEPES; pH was adjusted to 7.2 with KOH. The pipette solution for sodium channel current recording contained (in mM): 10 NaF, 110 CsF, 20 CsCl, 10 EGTA and 10 HEPES; pH was adjusted to 7.35 with CsOH. The extracellular solution contained (in mM) 140 NaCl, 5.4 KCl, 1.8 CaCl_2_, 0.5 MgCl_2_, 0.33 NaH_2_ PO_4_, 5.5 glucose, and 5.0 HEPES; pH was adjusted to 7.4 with NaOH. *I*_Kr_ blocker E-4031 (Wako, JAPAN) was dissolved in distilled water to yield 5 mM stock solution. The *I*_Ks_ blocker HMR1556 (Aventis Pharma Deutchland GmbH) was dissolved in dimethyl sulfoxide (DMSO, Sigma) to yield a 10 mM stock solution. Isoproterenol (Sigma) was dissolved in distilled water (containing 1 mM ascorbic acid) to yield a 10 mM stock solution and was kept in the dark at 4 °C.

### Electrophysiological recordings and data analysis

Forty eight hours after transfection, cells attached to a glass coverslip were transferred to a 0.5-ml bath chamber perfused with extracellular solution and maintained at 25 °C (for *I*_Ks_ and *I*_Kr_) or at 22–23 °C (for *I*_Na_). Patch-clamp experiments were conducted on GFP positive cells. Whole-cell membrane currents were recorded with an EPC-8 patch-clamp amplifier (HEKA, Lambrecht, Germany) and a resistance of 2.5 to 3.5 MΩ (*I*_Ks_ and *I*_Kr_) or 1.5 to 2.0 MΩ (*I*_Na_) electrodes.

Currents were evoked by depolarizing voltage-clamp steps given from a holding potential of −80 mV (*I*_Ks_ and *I*_Kr_) or −120 mV (*I*_Na_) to various test potentials. Amplitudes of both *I*_Ks_ and *I*_Kr_ were determined by measuring the amplitude of tail current. All currents were normalized to the cell membrane capacitance to obtain current densities (pA/pF). The protocols used for the assessment of the voltage dependence of activation/inactivation and recovery from inactivation are provided as insets in the relevant figures. Voltage-dependence of activation/inactivation were evaluated by fitting the *I*-*V* relation of currents to a Boltzmann as previously described^12^. The deactivation kinetics of *I*_Ks_ after depolarization and the recovery from inactivation data of *I*_Kr_ were determined by a single exponential equation: Y(t) = A_0_ + A exp(−t/τ). The deactivation kinetics of *I*_Kr_ after depolarization, the recovery from inactivation of *I*_Na_ and decay phase of *I*_Na_ data were fitted with a bi-exponential function of the form: Y(t) = A_0_ + A_f_ exp(−t/τ_f_) + A_s_ exp(−t/τ_s_), where A_f_ and A_s_ are the fractions of fast and slow relevant components, respectively. The persistent inward (late) *I*_Na_, a hallmark of biophysical abnormality in LQT3, was determined as the tetrodotoxin (TTX, 30 µM)-sensitive current measured after 800 ms of depolarization at −20 mV.

### Immunocytochemistry

Forty-eight hours after transfection, CHO cells were fixed with 4% paraformaldehyde in phosphate-buffered saline (PBS, pH 7.4) for 20 min and permeabilized with 0.2% Triton X-100 in PBS (PBST) for 10–20 min at room temperature. Cells were blocked with 10% Blocking One (Nacalai, Japan) in PBST for 30 min and then incubated overnight at 4 °C with rabbit polyclonal anti-Kv7.1 antibody (1:5000, Santa Cruz Biotechnology, Inc., Santa Cruz, CA, USA) and goat polyclonal anti-Kv11.1 antibody (1:1000, Santa Cruz Biotechnology, Inc., Santa Cruz, CA, USA). Following incubation, cells were labeled with an AlexaFluor^®^ 488-conjugated donkey anti-rabbit IgG (Molecular Probes, Eugene, Oregon) at 1:400 dilution for KCNQ1 or with an AlexaFluor^®^ 568-conjugated donkey anti-goat IgG (Molecular Probes, Eugene, Oregon) at 1:400 dilution for hERG. Nuclei were stained with 4′-6-diamino-2-phenylindole (DAPI). Immunofluorescence stained cells were captured using a confocal laser scanning system Clsi (Nikon) on an Eclipse TE2000-E inverted microscope (Nikon). In the present study, if a cell showed KCNQ1 expression, above 90% of such cells simultaneously showed hERG expression in co-expression experiments.

### Statistical analysis

All data are expressed as mean ± SE, with the number of experiments in parentheses. Statistical comparisons were analyzed using unpaired Student *t*-test or 1-way ANOVA with Newman-Keuls *post hoc* test. A *P* value of < 0.05 was considered statistically significant.

### Data Availability

The datasets generated during and/or analysed during the current study are available from the corresponding author on reasonable request.

## Electronic supplementary material

Dataset 1
