# Supplementary material for: A hERG mutation E1039X produced a synergistic lesion on IKs together with KCNQ1-R174C mutation in a LQTS family with three compound mutations
Source: Sci Rep. 2018 Feb 15;8:3129. doi: 10.1038/s41598-018-21442-6 (PMC5814447; doi:10.1038/s41598-018-21442-6)
Supplement: Supplementary file 1 — Dataset 1 [file 41598_2018_21442_MOESM1_ESM.doc]

***Supplementary Materials***

**A hERG mutation E1039X produced a synergistic lesion on *I*_Ks_ together with KCNQ1-R174C mutation in a LQTS family with three compound mutations**

Jie Wu, PhD^1,2,3^, Yuka Mizusawa, MD^2^, Seiko Ohno, MD, PhD^2^, Wei-Guang Ding, MD, PhD^3^, Takashi Higaki, MD, PhD^4^, Qi Wang, PhD^2^, Hirohiko Kohjitani, MD^5^, Takeru Makiyama, MD, PhD^5^, Hideki Itoh, MD, PhD^2^, Futoshi Toyoda, PhD^3^, Andrew F James, PhD^6^, Jules C Hancox, PhD^6^, Hiroshi Matsuura, MD, PhD^3^, Minoru Horie, MD, PhD^2,*^

^1^ Department of Pharmacology, Medical School of Xi’an Jiaotong University, Xi’an, Shaanxi 710061, China

^2^ Department of Cardiovascular and Respiratory Medicine, Shiga University of Medical Science, Shiga, Japan

^3^ Department of Physiology, Shiga University of Medical Science, Shiga, Japan

^4^ Department of Pediatrics, Ehime University School of Medicine, Ehime, Japan

^5^ Department of Cardiovascular Medicine, Kyoto University Graduate School of Medicine, Kyoto, Japan

^6^ School of Physiology, Pharmacology and Neuroscience, University of Bristol, Bristol, United Kingdom

**Correspondence author:**

Minoru Horie, MD, PhD

Department of Cardiovascular and Respiratory Medicine, Shiga University of Medical Science, Seta Tsukinowa-cho, Otsu, 520-2192, Japan

Phone: +81-77-548-2213, Fax: +81-77-543-5839

E-mail: [horie@belle.shiga-med.ac.jp](mailto:horie@belle.shiga-med.ac.jp)

Total word count: 896

Figures: 6

For analyzing the effect of mutations on the cardiac action potential duration (APD), we conducted an in silico study by using the human ventricular cell (HuVEC) model^1^. Because the patients’ genotype in the present study is rather complicated and different to reconstitute totally. In order to mimic heterozygous state of patients in silico study, we presume that a patient would have different simulation formations: WT, mutant and then WT-mutant, in which an assumption is made that a 50:50 mix of WT and mutant formulations would mimic the heterozygous state of the patient. So we use an intermediate formulation to do the simulation in silico analysis. The pacing cycle length is 1000 ms and the serum potassium concentration is 4.5 mmol/L.

The computer simulation result shows that APD_90_ of human ventricular cell is 335.1 ms (Fig. 1) under the control condition (WT). However, the APD is prolonged to 365.4 ms (Fig. 2) by KCNQ1-R174C, hERG-E1039X and SCN5A-E428K mutations. Furthermore, if the interaction between KCNQ1-R174C and hERG-E1039X is taken into consideration, the APD_90_ will be further prolonged to 376.5 ms (Fig. 3). The above result is consistent with the function analysis of “*I*_Ks_” + “*I*_Kr_” channels co-expressed in our mammalian expression system, which provides a mathematical model support for the present study that two *α* subunits might produce a synergistic lesion in cardiac channel function.

Meanwhile, we find that APDs in mutant conditions (Fig. 5 and Fig. 6) are easier affected by serum potassium concentrations than those under control condition (Fig. 4), especially when the interaction between KCNQ1-R174C and hERG-E1039X is taken into consideration (Fig. 6). These results also provide a support for the above suggestion that KCNQ1-R174C and hERG-E1039X APD might produce a synergistic lesion in cardiac channel function.

**References**

1. Himeno Y, et al. A human ventricular myocyte model with a refined representation of excitation-contraction coupling. *Biophys J.* **109**: 415-427 (2015).


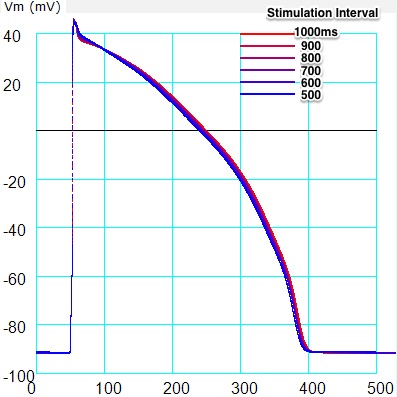
 **Time (ms)**

| **Pacing Cycle Length (ms)** | **APD_90_**  **(ms)** |
| --- | --- |
| **1000** | 335.1 |
| **900** | 333.5 |
| **800** | 332.6 |
| **700** | 331.8 |
| **600** | 331.0 |
| **500** | 325.0 |

**Figure 1.** Effects of KCNQ1-WT, hERG-WT and SCN5A-WT on human ventricular action potential durations (APDs) in HuVEC model.

**Left**: APDs; **Right**: the table lists different pacing cycle lengths and related APDs. Serum potassium concentration: 4.5 mmol/L


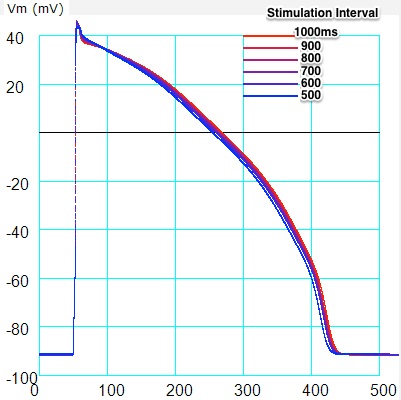


| **Pacing Cycle Length (ms)** | **APD_90_**  **(ms)** |
| --- | --- |
| **1000** | 365.4 |
| **900** | 363.3 |
| **800** | 362.2 |
| **700** | 361.3 |
| **600** | 356.4 |
| **500** | 351.7 |

**Time (ms)**

**Figure 2.** Effects of KCNQ1-R174C, hERG-E1039X and SCN5A-E428K mutations on the human ventricular action potential duration (APD) in HuVEC model, without consideration of the interaction between KCNQ1-R174C and hERG-E1039X.

**Left**: APDs; **Right**: the table lists different pacing cycle length and related APDs. Serum potassium concentration: 4.5 mmol/L


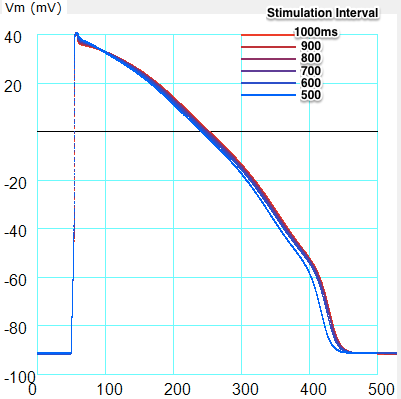


| **Pacing Cycle Length (ms)** | **APD_90_ (ms)** |
| --- | --- |
| **1000** | 376.5 |
| **900** | 375.6 |
| **800** | 374.9 |
| **700** | 374.2 |
| **600** | 371.5 |
| **500** | 362.2 |

**Time (ms)**

**Figure 3.** Effects of KCNQ1-R174C, hERG-E1039X and SCN5A-E428K mutations on the human ventricular action potential duration (APD) in HuVEC model, with consideration of the interaction between KCNQ1-R174C and hERG-E1039X.

**Left**: APDs; **Right**: the table lists different pacing cycle lengths and related APDs. Serum Potassium Concentration: 4.5 mmol/L.


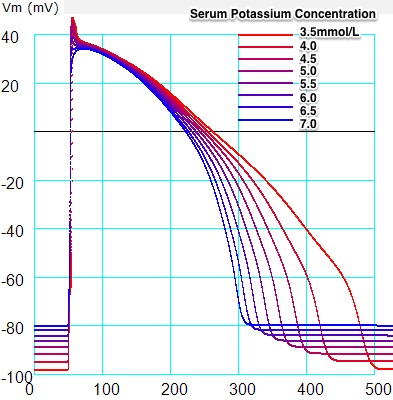


| **Serum K^+^ Concentration (mmol/L)** | **APD_90_ (ms)** |
| --- | --- |
| **3.5** | 429.6 |
| **4.0** | 369.3 |
| **4.5** | 335.1 |
| **5.0** | 309.4 |
| **5.5** | 285.7 |
| **6.0** | 268.4 |
| **6.5** | 242.8 |
| **7.0** | 226.8 |

**Time (ms)**

**Figure 4.** Effects of serum potassium concentrations on the human ventricular action potential duration (APD) in control (WT) HuVEC model.

**Left**: APDs; **Right**: the table lists different serum potassium concentrations and related APDs. Pacing cycle length: 1000 ms.


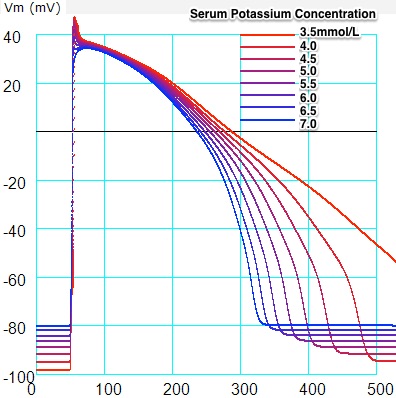


| **Serum K^+^ Concentration (mmol/L)** | **APD_90_ (ms)** |
| --- | --- |
| **3.5** | 526.5 |
| **4.0** | 428.7 |
| **4.5** | 365.4 |
| **5.0** | 332.9 |
| **5.5** | 307.1 |
| **6.0** | 286.8 |
| **6.5** | 255.9 |
| **7.0** | 240.7 |

**Time (ms)**

**Figure 5.** Effects of serum potassium concentrations on the human ventricular action potential duration (APD) in mutant (KCNQ1-R174C, hERG-E1039X and SCN5A-E428K) HuVEC model, without consideration of the interaction between KCNQ1-R174C and hERG-E1039X.

**Left**: APDs; **Right**: the table lists different serum potassium concentrations and related APDs. Pacing cycle length: 1000 ms.


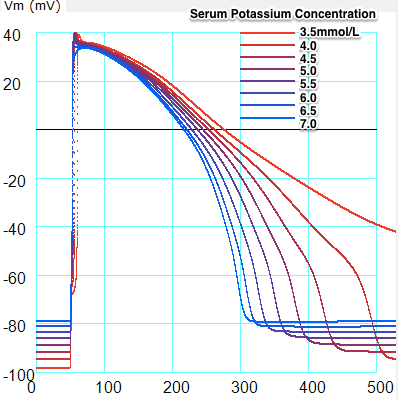


| **Serum K^+^ Concentration (mmol/L)** | **APD_90_ (ms)** |
| --- | --- |
| **3.5** | 669.0 |
| **4.0** | 447.8 |
| **4.5** | 376.5 |
| **5.0** | 335.9 |
| **5.5** | 306.1 |
| **6.0** | 282.1 |
| **6.5** | 255.0 |
| **7.0** | 236.4 |

**Time (ms)**

**Figure 6.** Effects of serum potassium concentrations on the human ventricular action potential duration (APD) in mutant (KCNQ1-R174C, hERG-E1039X and SCN5A-E428K) HuVEC model, with consideration of the interaction between KCNQ1-R174C and hERG-E1039X.

**Left**: APDs; **Right**: the table lists different serum potassium concentrations and related APDs. Pacing cycle length: 1000 ms.
